# Fused Filament Fabrication of NiTi Components and Hybridization with Laser Powder Bed Fusion for Filigree Structures

**DOI:** 10.3390/ma14164399

**Published:** 2021-08-06

**Authors:** Johannes Abel, Anne Mannschatz, Robert Teuber, Bernhard Müller, Omar Al Noaimy, Sebastian Riecker, Juliane Thielsch, Björn Matthey, Thomas Weißgärber

**Affiliations:** 1 Fraunhofer Institute for Ceramic Technologies and Systems (IKTS), Winterbergstraße 28, 01277 Dresden, Germany; anne.mannschatz@ikts.fraunhofer.de (A.M.); bjoern.matthey@ikts.fraunhofer.de (B.M.); 2 Fraunhofer Institute for Manufacturing Technology and Advanced Materials (IFAM), Winterbergstraße 28, 01277 Dresden, Germany; robert.teuber@ifam-dd.fraunhofer.de (R.T.); sebastian.riecker@ifam-dd.fraunhofer.de (S.R.); thomas.weissgaerber@ifam-dd.fraunhofer.de (T.W.); 3 Fraunhofer Institute for Machine Tools and Forming Technology (IWU), Nöthnitzer Str. 44, 01187 Dresden, Germany; bernhard.mueller@iwu.fraunhofer.de (B.M.); omar.al.noaimy@iwu.fraunhofer.de (O.A.N.); juliane.thielsch@iwu.fraunhofer.de (J.T.)

**Keywords:** smart material, Shape Memory Alloy, superelastic alloy, pseudoelastic alloy, nickel–titanium, nitinol, fused filament fabrication (FFF), Additive Manufacturing (AM), hybridization, Laser Powder Bed Fusion (LPBF)

## Abstract

The present study introduces an approach to the powder metallurgical shaping of a pseudo-elastic nickel–titanium (NiTi 44 alloy) combining two different Additive Manufacturing (AM) processes, namely fused filament fabrication (FFF) and Laser Powder Bed Fusion (LPBF), by manufacturing filigree structures on top of sintered FFF parts. Both processes start with commercial gas atomized NiTi powder, which is fractionated into two classes. Using the fine fraction with particle sizes <15 µm, robust thermoplastic filaments based on a non-commercial binder system were produced and processed to different auxetic and non-auxetic geometries employing a commercial standard printer. FTIR analysis for thermal decomposition products was used to develop a debinding regime. After sintering, the phase transformation austenite/martensite was characterized by DSC in as sintered and annealed state. Precipitates resulting from residual impurities were detected by micrographs and XRD. They led to an increased transformation temperature. Adjusting the oxygen and carbon content in the alloy remains a challenging issue for powder metallurgical processed NiTi alloys. Filigree lattice structures were built onto the surfaces of the sintered FFF parts by LPBF using the coarser powder fraction (15–45 µm). A good material bond was formed, resulting in the first known NiTi hybrid, which introduces new production and design options for future applications.

## 1. Introduction 

Recent years have witnessed a growing interest in NiTi Shape Memory Alloy (SMA) due to its combination of outstanding functional features, such as shape memory and pseudoelasticity/superelasticity [1,2]. As a result of these unique properties, NiTi (also known as Nitinol) has become a broadly used material in different applications, ranging from sporting goods, actuators, automotive industries and electrical safety devices to medical tools and aerospace uses [3]. However, the production of complex NiTi structures using conventional processing techniques (e.g., machining, laser cutting, electron beam welding) has faced obstacles. The major reasons for the processability can be ascribed to the high ductility, strong work hardening, high toughness and compositional sensitivity of NiTi alloys [4]. Therefore, to overcome these difficulties, a solution is needed, which has been found by using Additive Manufacturing (AM) processes as an excellent alternative process to fabricate complex NiTi structures with adjustable or even programmable characteristics. 

Due to their inherent properties, so called ‘programmable materials’ have the potential for high functional integrability with low system complexity at the same time, since their internal design enables the adoption of functions of complete systems. This is achieved by a specific inner structure of these programmable materials, which allows us to control and reversibly change their properties and behavior—not only globally but also with local variation of functionality. To fully exploit the potential of programmable materials, the utilization of smart materials in combination with optical and mechanical metamaterials is required. Additive Manufacturing is a key enabling technology for programmable materials, according to the Fraunhofer Cluster of Excellence Programmable Materials (CPM), due to its unique potential to combine the processing of smart materials with superior properties with design freedom, e.g., for metamaterials based on small-scale unit cells [5].

Laser Powder Bed Fusion (LPBF or PBF-LB) technology is being used to manufacture highly complex and extremely filigree lattice structures out of a NiTi alloy—a smart material that can show shape memory as well as pseudoelastic behavior depending on its composition. The development and adaption of the LPBF process has enabled AM of programmable metal materials. While LPBF is the AM technology of choice to manufacture small and filigree parts and features, it has a clear disadvantage in terms of cost, when it comes to larger and more compact or bulky components of limited three-dimensional complexity. For faster AM at lower cost, fused filament fabrication (FFF) technology has been identified as a viable alternative route for AM of NiTi structures, especially when it comes to larger, bulkier and less complex parts, such as high-performance actuators. Filament-based 3D printing of NiTi has high industrialization potential. This is mainly because it utilizes low-cost 3D printing equipment that is widely used worldwide, from private homes through Fab Labs and Maker Spaces to 3D print shops and industrial 3D printing factories. This means that it demands a rather low level of expertise to operate this equipment. Secondly, filament-based raw material implies further cost-saving potential compared to powder bed-based AM technologies. Firstly, fine powders below 10 µm which remain as a non-usable or waste powder fraction for LPBF can be used for the filament production. Fine powders are often formed during metal powder production and are separated from the target fraction e.g., by sieving since they exhibit a poor flowability, thus allowing for a significantly lower raw material cost for FFF. Secondly, much cheaper water-atomized powders can be utilized for FFF instead of gas-atomized ones.

However, this cost-saving potential needs to be considered in context with the additional cost for FFF compared to LPBF—in terms of filament preparation (compounding of metal powder with binder plus filament extrusion) and in terms of the further thermal processing of FFF printed components (debinding, sintering). These steps are necessary to achieve a highly dense microstructure compared to PBF, which creates fully dense parts in only one process step, directly from metal powder. Filament manufacturing for FFF allows the usage of “scrap” powder emerging in gas atomization for LPBF, as well as a high solid loading (>60 vol.%) of metallic particles for a high metal density of green FFF parts. Another major advantage of filament-based AM compared to powder bed fusion is the potential for multi-material use via switching between several filament feedstocks during manufacturing. Further advantages of FFF over LPBF AM for NiTi have been previously considered, such as difficulties in LPBF processing, Ni content and transformation temperature volatility and the formation of undesirable phases during melting and solidification [6].

Although FFF processes provide a limited level of complexity in the third dimension (Z) using no support structure, they allow for rather high complexity in the first two dimensions (X and Y) in terms of minimum feature size (wall thickness) and in terms of geometric intricacy, being able to manufacture highly complex components, e.g., auxetic structures. In addition, FFF also qualifies for the AM of programmable metamaterials to a certain extent. Expanding the boundaries even further, the hybridization of FFF and LPBF AM bears high potential for affordable NiTi components of size and complexity, combining the main advantages of both AM technologies, e.g., for large, rather bulky or compact parts with complexity limited to certain part features or localities. 

### 1.1. State-of-the-Art Fused Filament Fabrication (FFF) of Particle Filled Filaments

FFF is a Material Extrusion (MEX) AM method using a thermoplastic filament as feedstock standardized in ISO/ASTM 52900:2015. It was established in AM of thermoplastics using, e.g., polylactide (PLA), acrylonitrile butadiene styrene (ABS) or polycarbonate (PC) filaments, which are molten in a heated nozzle and are formed when the material is in a soft state, on a building plate, where it is solidified due to cooling. This method is adopted for powder metallurgical manufacturing by using the same thermoplastic filaments but filling them with ceramic, metal or glass particles prior to AM deposition [7,8,9,10]. This approach to additively shape ceramic and metal materials has been used since the 1990s and has experienced a significant increase in popularity in recent years [11,12,13,14,15,16,17].

After shaping the green body, a thermal treatment is necessary in order to decompose the thermoplastic organics and densify the microstructure by a sintering process. These two process steps are the most expensive and most attention-demanding, especially when it comes to oxidation-sensitive powders. FFF is known as a process that leads to high surface roughness compared to other AM processes, such as Vat Photopolymerization (VPP) [18]. The surface quality can be enhanced by green machining or using small nozzles, which leads to a reduced staircase effect due to the reduced layer heights. Thereby, small components can be produced with satisfying surface roughness by using nozzle diameters of 150 µm [19]. Moreover, FFF is able to cover a broad field of application since both small and large components can be manufactured. Using low-cost, off-the-shelf equipment and the growing commercial material portfolio of particle-filled filaments, FFF offers an economical alternative in terms of green part manufacturing. 

Recently, Carriera et.al. [6] published a contribution that investigated the optimal heat treatment of NiTi specimens manufactured by means of FFF containing different thermoplastic polymers and using different atmospheres such as vacuum or argon. Since thermoplastic filaments contain a high ratio of polymers (~35–60 vol.%), the formation of carbides must be considered, which is one of the most significant challenges of using NiTi powders in a thermoplastic approach.

### 1.2. State-of-the-Art Laser Powder Bed Fusion (LPBF) of Metals and Hybridization with Other Fabrication Methods

LPBF is a direct AM method established in prototyping and low volume as well as individualized production of metal components, especially for the aerospace [20], medical [21] energy [22] and other [23] sectors. It is historically also known as Selective Laser Melting (SLM), Laser Beam Melting (LBM), Direct Metal Laser Sintering (DMLS) and many other terms, most of them branded by OEM system manufacturers. LPBF of functional or smart materials includes magnetic materials, e.g., NdFeB [24], and Shape Memory Alloys, e.g., NiTi [25,26]. With LPBF having major advantages in the manufacturing of delicate, low-mass and small-sized components with superior mechanical properties at competitive prices compared to conventional manufacturing, a major strategy for LPBF application can be found within super lightweight lattice structures with excellent stiffness-to-weight ratio [27]. Another major advantage of LPBF is the hybridization potential, allowing us to “print” with LPBF onto conventionally or otherwise manufactured base bodies or substrates, a technique that is widely used in AM tooling applications [28]. In comparison to other AM technologies, especially Material Extrusion and binder jetting technology (BJT) AM methods, the build ratio (volume generated per time) of LPBF is rather low, but in most cases, LPBF parts can be applied directly without the need for post-treatment such as debinding and sintering.

## 2. Materials and Methods

### 2.1. Powder Material and Binder

Within this study, an atomized intermetallic NiTi44 powder from Nanoval (Nanoval GmbH & Co.KG, Berlin, Germany) was used to obtain pseudoelastic properties in the sintered parts. To investigate the particle shape, a FESEM Zeiss Ultra 55 (Carl Zeiss AG, Oberkochen, Germany) was utilized. The total atomized quantity was classified into two fractions. For the FFF process, the fine powder fraction <15 µm was used, which is normally sorted out for Laser Powder Bed Fusion since the recoating properties can be affected negatively [29]. For the LPBF hybridization process, the 15–45 µm fraction was used. Initial powder impurities C, O, N were determined with ONH 836, TCH 600 and CS 230, Leco Instrumente GmbH, Germany, and were found to be: 0.045 wt.% C, 0.155 wt.% O, <0.005 wt.% N. For evaluation of the present phases, XRD measurements on a D8 Advance (Bruker AXS, Karlsruhe, Germany) using Cu-Ka radiation in the range of 5–100° 2θ were performed. The qualitative phase analysis was carried out using Diffrac.EVA (Bruker AXS, Karlsruhe, Germany) and a PDF-2021 database (ICDD).

A thermoplastic, non-commercial, polyamide-based binder system (Inmatec Technologies GmbH, Reihnbach, Germany) with excellent properties for filament preparation was applied. The binder system exhibits properties such as flexibility combined with high strength. Due to its low viscosity, it provides good processing properties in the solid loading range of 45–65 vol.% for metals and ceramics and can be used in standard FFF machines. The polymer requires a two-step debinding process, consisting of solvent pre-debinding in acetone and thermal decomposition in a powder-specific atmosphere (e.g., oxidizing, reducing or inert). Since titanium exhibits a high oxidation tendency, argon was used in this study to prevent this phenomenon during debinding. In addition to the typical carbon in organics, it must be noted that polyamide has oxygen in the molecular chain, which may not be reduced in thermal debinding. This contamination can lead to unwanted oxidation of the NiTi. Therefore, additional XRD was applied in the sintered state to evaluate the impurities after the complete process chain compared with the initial powder state.

### 2.2. Manufacturing of Thermoplastic Filament

Initial compounding tests were performed at 175 °C in a torque rheometer (Plastograph, Brabender GmbH & Co.KG, Duisburg, Germany) with a kneading chamber volume of 50 cm^3^. The kneading behavior for solid loadings between 55 vol.% and 63 vol.% was evaluated; 63 vol.% (91 wt.%) proved to be suitable for further development. For manufacturing the filament, four steps were undertaken. Powder and binder were premixed in a heated kneader (Planetron HKV5, IKA, Staufen im Breisgau, Germany) under argon circulation to minimize oxygen uptake. The binder coats the powder particles, which covers their surface and prevents demixing during the feeding. The premix was fed into a twin-screw extruder (KETSE20/40, Brabender, Duisburg, Germany) to produce an evenly homogenized feedstock. After three passes, the resulting pressure was constant and the feedstock was extruded through a 3.5 mm nozzle and granulated with a rotating knife, to produce 4 mm long cylindrical granules. A homogenous granule size distribution, without dust, is necessary to ensure steady material flow in the single-screw extruder (D30, Brabender, Duisburg, Germany). After establishing suitable production parameters (feeding, screw rotation, velocity of cooling conveyer, velocity of winder), the filament was extruded through a 1.9 mm nozzle at 120 °C. By slightly stretching the extrudate, the filament diameter could be calibrated to the nominal value of 1.75 mm.

### 2.3. Additive Manufacturing by Means of FFF

The basis for AM is a CAD data file generated in conventional CAD programs. These data are transferred as an STL file to the slicing program, where they are parameterized according to the print strategy and device-specific aspects. For slicing, the commercial software Simplify3D^®^ Version 4.1.2 (Simplify3D^®^, Blue Ash, OH, USA) was used.

For AM of the samples, a standard printer Prusa i3 MK3S+ (Prusa Research, Prague, Czech Republic) with a standard brass nozzle was used. Manufacturing parameters after parameter adaption are presented in Table 1.

To demonstrate the system openness of the filament, print tests were performed on a Hage 140L (Hage 3D GmbH, Obdach, Austria), which has a direct belt drive and a direct driven Renkforce RF100 (Conrad Electronic SE, Hirschau, Germany). The results showed that the filament does not cause any problems when manufacturing parts with these commercially available standard machines.

### 2.4. Solvent Debinding

Debinding was a two-step process. In the first step, 73 wt.% of the initial binder was extracted by acetone at 35 °C for 48 h using solvent debinding equipment (MDU 30, DesbaTec Anlagentechnik, Sulzbach am Main, Germany). It is important to ensure that slow drying of the parts takes place to prevent drying cracks during contraction of the backbone polymer swollen in the solvent. This is one critical step in the applied process chain.

After complete drying, the samples were thermally debound to degrade the residual higher molecular polymers.

### 2.5. Thermal Debinding and Sintering

To analyze the debinding behavior, a heat treatment was carried out under argon (Ar 6.0) at 60 mbar with a constant heating rate of 3 K/min between 20 °C and 1200 °C, and the gas composition was recorded in situ by means of Fourier transform infrared spectroscopy (FTIR) (Fraunhofer IFAM, Dresden, Germany). In order to collect in-situ data during thermal debinding, a special setup and data processing were applied. In this setup, a light beam passed through two infrared-transmissive windows and was lead through the furnace chamber, above the sample material. On the other side of the furnace, the spectrum of the light beam was measured with an external FTIR detector. The area under the characteristic peak of each gas species was used as a measure of absorption. This quantity is a relative measure for the concentration of the respective gas species in the atmosphere. 

The results obtained in the FTIR analysis provide, among others, information about holding times for the thermal debinding. Based on this information, the heat treatment regime was adjusted with holding times of 60 min for temperatures 330 °C, 370 °C, 450 °C. The temperature was then further increased to a sintering temperature of 1180 °C and held for 240 min.

The heat treatment experiments (thermal debinding, sintering) were carried out in an iso-furnace (MUT Advanced Heating GmbH, Jena, Germany). Argon with a purity ≥99.9999% was used as the process gas. Thermal debinding was performed under a pressure of 60 mbar argon. Sintering was run under high vacuum at 1 × 10^−4^–4 × 10^−5^ mbar argon.

Following thermal debinding and sintering, homogenization annealing was performed using a laboratory furnace (Nabertherm GmbH, Lilienthal, Germany) and a subsequent heat treatment on a further laboratory furnace from Arnold Schröder Industrieöfen GmbH, Flörsheim am Main, Germany. These two downstream heat treatment steps were carried out with the aim of specifically adjusting the transformation temperatures for the effect of pseudoelasticity. For homogenization annealing, samples were enclosed in a quartz glass tube under argon and sealed airtight. This was followed by heating up to 950 °C. The temperature was kept constant for 5 h. Subsequently, the samples were removed from the furnace, the quartz glass tube was broken open and the samples quenched in water. 

The second subsequent heat treatment step was a heating step to a defined temperature, which was held for one hour. In two series of experiments, heating was performed once to 450 °C and once to 550 °C. Both series of tests were carried out under air.

### 2.6. Hybridization of FFF and LPBF

A NiTi sample of 17 × 17 × 10 mm^3^ fabricated via FFF was used as a substrate for the hybridization process. Therefore, the FFF sample was cleaned with acetone at 56 °C for 30 min. The specimen was flat-ground to ensure plane-parallel clamping in the laser device. The LPBF process was performed utilizing an M2 system (Concept Laser GmbH, Lichtenfels, Germany), equipped with a 400 W continuous-wave, diode-pumped fiber laser (wavelength = 1070 nm, laser focus diameter = 100 μm). To reduce the contamination with oxygen, LPBF processing was run under a high-purity argon atmosphere. For hybridization, the aforementioned gas-atomized powder of NiTi44 with particle sizes of 15–45 μm was used to build the filigree NiTi body-centered cubic (bcc) lattice structure (with dimensions 12 × 12 × 6 mm^3^) on the NiTi FFF sample. The LPBF processing parameters used to manufacture the bcc lattice structures were: laser power 200 W, layer thickness 25 µm, line pseudo-P scanning (vector length= 0.1 mm) and scanning speed 500 mm/s.

### 2.7. Characterization of Components

Printed samples were scanned by X-ray radiography using a CT Compact (Procon X-ray, Sarstedt, Germany) with a 180 kV microfocus tube and a flat panel detector with 5888 × 4600 pixel resolution. The pixel size was 50 µm, binned 2 × 2 for the measurement. The samples were scanned with 130 kV and 150 µA and an exposure time of 500 ms.

The temperature-dependent phase change of Nitinol was measured by differential scanning calorimetry (DSC) (DSC 204F1 Phoenix, NETZSCH-Gerätebau GmbH, Selb, Germany). The measurements were carried out under a nitrogen atmosphere (40 mL/min–60 mL/min) in a temperature range between −110 °C and 160 °C. The heating rates were 10 K/min in each case. Oxygen and nitrogen impurities were determined by IR absorption and thermal conductivity measurement using tin capsule and melting accelerator Ni basket (ONH 836 and TCH 600, Leco Instrumente GmbH, Mönchengladbach, Germany). The carbon and sulfur impurities were detected by IR absorption after combustion in the induction furnace (measuring gas: oxygen) (CS 230, Leco Instrumente GmbH, Mönchengladbach, Germany). Lecocel II and Fe chips were used as aggregates. 

Sintered samples were cut and polished for cross-section analysis via a reflected light microscope (Axio Observer, Carl Zeiss Microscopy Deutschland GmbH, Berlin, Germany).

The hybrid sample was additionally prepared for microstructure analysis by wetting the surface with a solution of 10 mL H_2_O, 0.2 mL HNO_3_, 0.2 mL HF (grain boundary etching after Kroll) for 1–2 min.

EDS mapping of the sintered component was carried out by a XMax150 (Oxford Instruments, Abingdon, UK), providing information about involved elements.

## 3. Results

**Figure 1:**
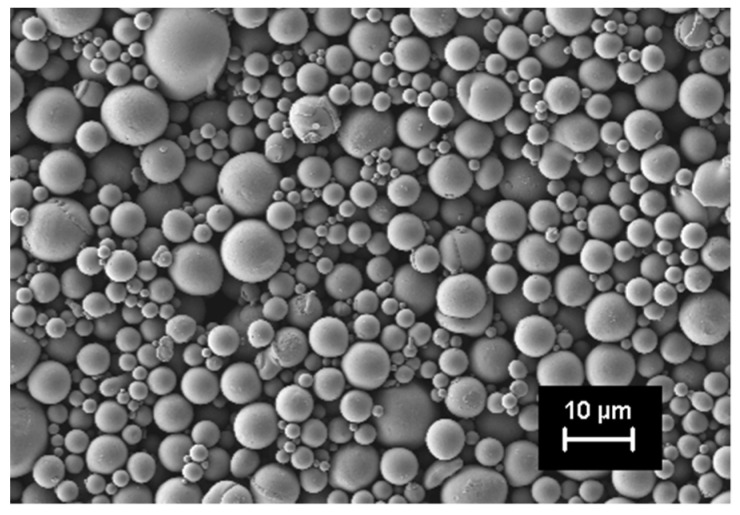
SEM of gas-atomized NiTi 44 powder with particle size <15 µm in initial state for filament production.

The initial powder was investigated by SEM, revealing spherical particles, which was evident for gas atomized powders, as shown in Figure 1.

This shape ensures good flowability in the feedstock melt. The powder bulk density measured was 6.45 g/cm^3^.

**Figure 2:**
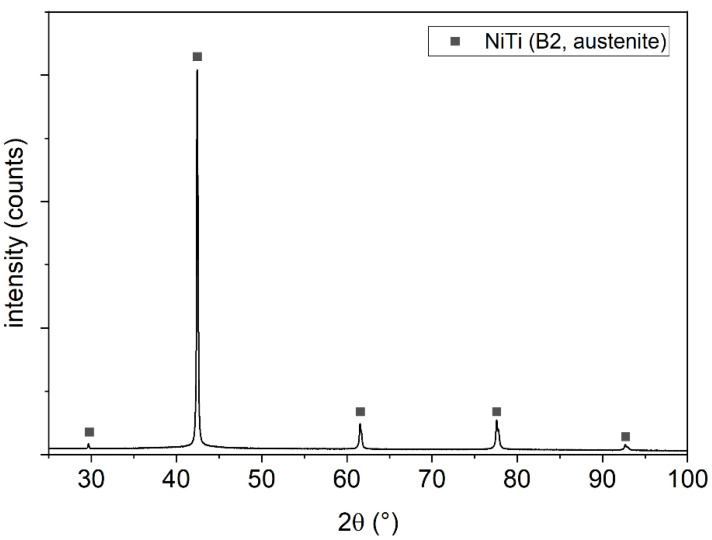
XRD of the initial powder showing only the austenitic B2 phase of NiTi.

The XRD pattern in Figure 2 displays the high-temperature cubic B2 phase (austenite, space group Pm-3m) in the initial powder only. This is beneficial to achieve pseudoelastic properties within the sintered parts.

**Figure 3:**
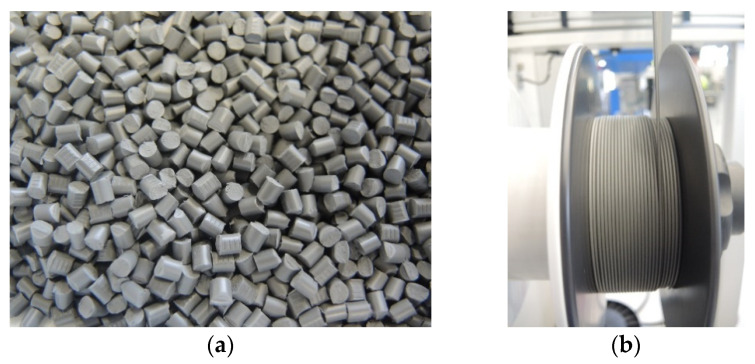
(**a**) Homogeneous feedstock granulated with a solid loading of 63 vol.%, (**b**) Continuous spooling of the 1.75 mm NiTi filament.

After melt compounding and granulation of the feedstock (Figure 3a), the filament with a diameter of 1.75 mm was continuously extruded within a tolerance range of 0.15 mm (Figure 3b).

**Figure 4:**
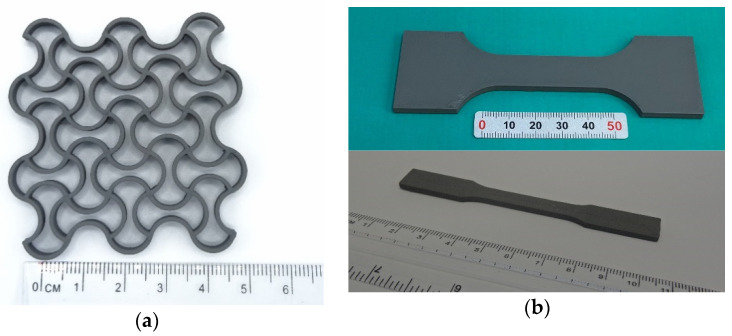
(**a**) Green part auxetic structure, (**b**) Tension rods.

The fabricated filament offers high flexibility combined with high strength at an exceptional high-volume loading of 63%. Using the parameters presented in Table 1, for example, the samples shown in Figure 4 were produced. It could be confirmed that the filament can be processed with a standard device without any problems.

**Figure 5:**
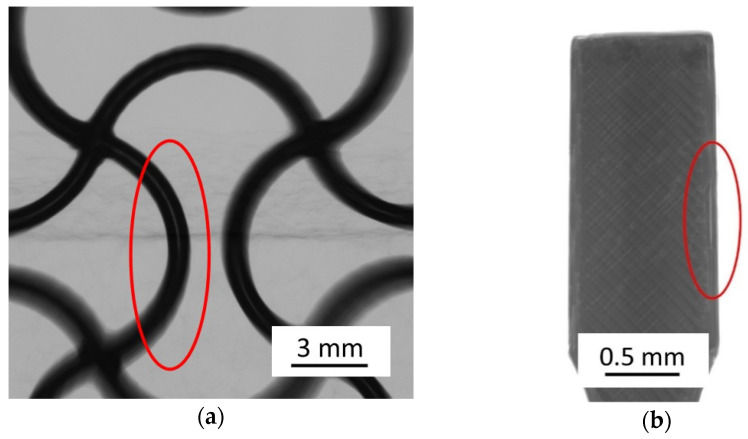
(**a**) Radiography of auxetic green body with adhesion defects between two outlines, (**b**) Radiography of tension rod with adhesion defects in infill +45°/−45°and between infill and outline.

Both the auxetic structure and the tension rod geometries were used for mechanical testing after sintering. Typical challenges such as the formation of voids by utilization of different infill strategies have been observed. By optimizing the manufacturing parameters, such phenomena can be minimized. Most voids or adhesion defects result from under-extrusion when material flow continuity is not maintained. In Figure 5, such defects are presented. Deflection points are particularly affected, since the nozzle undergoes a change in direction. Increasing the extrusion multiplier effectively decreases process-related voids when using standard software.

This phenomenon could be encountered in the future by modified path guidance and deposition strategies. Such approaches were already considered in the 1990s, but have not yet been significantly applied [30].

**Figure 6:**
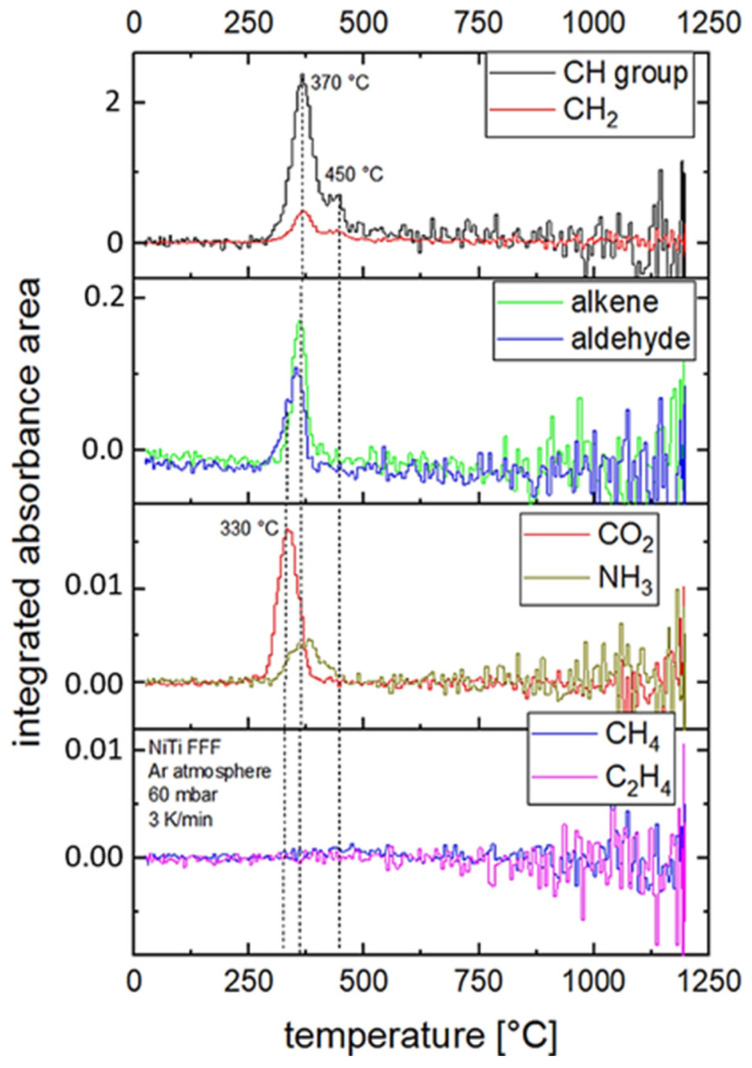
Gas phase analysis of printed FFF samples during heat treatment with argon at 3 K/min up to 1200 °C. The graphs show the integrated absorbance area of the respective gas species from the light spectrum (FTIR analysis) after the light beam has passed the furnace.

The solvent debinding and heat treatment were performed according to Section 2.4 and Section 2.5. The debinding behavior of the printed and pre-debound parts was investigated by means of in-situ gas phase analysis in a heat treatment test. The integrated absorbance area is plotted versus the furnace temperature for different organic components of the binder in Figure 6. This represents a relative measure of the amount of gas species outgassing from the green part during heat treatment depending on temperature. The absorbance area gives no information on absolute gas amounts. 

Based on the gas phase analysis, the temperature range for thermal debinding of the FFF samples could be specified in detail. The maxima in absorption are between 330 °C and 380 °C (CO_2_, alkenes, aldehydes, CH_2_, CH-groups, NH_3_) and at 450 °C (CH groups). No significant formation of CH_4_ and C_2_H_4_ was observed during the heat treatment experiment. The strongest signals in the spectra came from CH groups and CO_2_. The noise of the data increases with higher temperature. Above 500 °C, no significant debinding peak could be observed. This indicates a fully pyrolyzed sample.

The measurement was used to define the heating rates, temperature steps and holding times of the debinding regime. In order to avoid the formation of gas bubbles and cracks, as well as to minimize impurities, the first temperature step was set below the first peak and the heating rate was lowered in the mentioned range of thermal debinding. The parameters determined based on this measurement are described in Section 2.5. 

**Figure 7:**
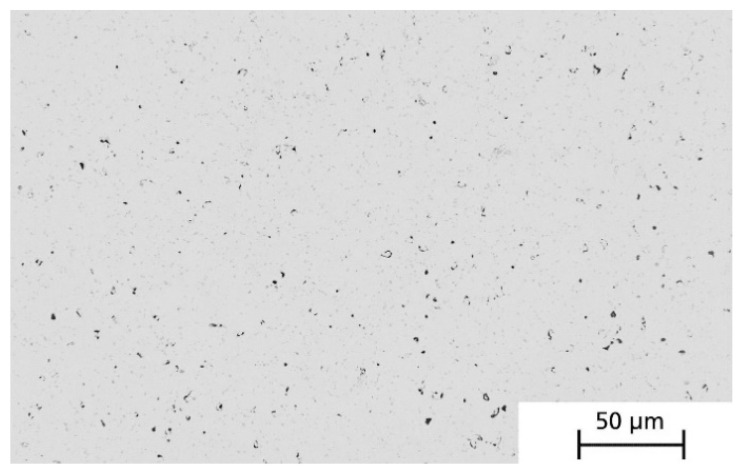
Micrograph of a sintered specimen within the printing strand with a residual porosity of 1.0 ± 0.7%.

The derived heat treatment parameters were tested in a sintering experiment on printed green specimens (box geometry, external dimensions 20 × 20 × 10 mm^3^). Figure 7 shows the micrograph of a sintered and ground specimen with homogeneously distributed porosity of approximately 1.0 ± 0.7%. The linear shrinkage was 11% and was found to be isotropic. However, larger pores could also be observed between the printing strands, which were evidently caused by deficient material. Depending on the component geometry, these printing defects can be prevented or greatly reduced by a locally adjusted extrusion rate depending on the printing path. 

After sintering, the specimens were dark in color and had a gray metallic appearance on the surfaces that were in contact with the sintering substrate.

**Figure 8:**
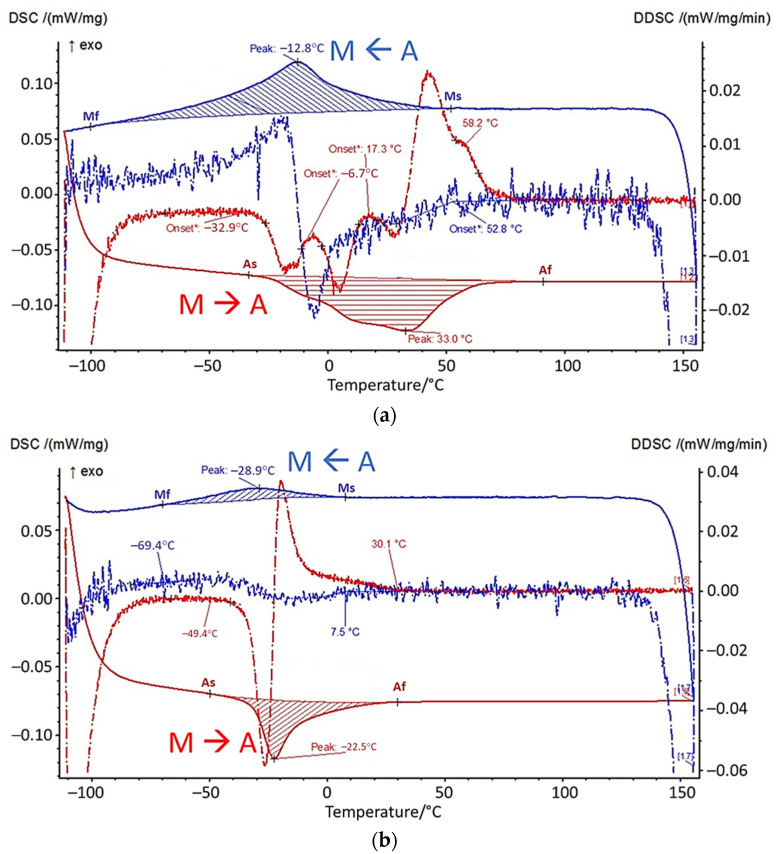
DSC measurements in the (**a**) as-sintered state, (**b**) after homogenization annealing and heat treatment at 550 °C for precipitation. Cooling is shown in blue, heating is shown in red.

The sintered samples were analyzed by DSC in different states to determine the phase transitions of austenite/martensite. Cylindrical samples with a diameter of 4 mm and a thickness of 1 mm were waterjet cut and used for the DSC measurements. These were always taken from the same area of the FFF-printed sample for the entire series of tests. The samples were analyzed both “as-sintered” and after homogenization annealing and subsequent heat treatment for precipitation at 450 °C and 550 °C, as described in Section 2.5. Figure 8 shows the DSC measurement in the as-sintered condition and the DSC measurement of the specimen heat-treated at 550 °C. The red lines indicate the heating phase, the blue lines the cooling phase during the measurement. The dashed lines show the derivative with respect to time.

The as-sintered sample shows a wide temperature range, in which the transition between martensite (M) and austenite (A) (and wise versa) takes place between 158 °C and −112 °C. In particular, the martensite-to-austenite transformation shows a strongly broadened peak with several local maxima, as can be seen in the DSC signal. The austenite finish temperature A_f_ is at approximately 90 °C. Homogenization and precipitation annealing at 550 °C leads to narrower peaks with transition temperature ranges of 77 °C (M<--A) and 80 °C (M-->A). The austenite finish temperature A_f_ is 30 °C.

After each heat treatment step, the samples were investigated regarding their impurities. For this purpose, the carbon, oxygen and nitrogen content were determined as described in Section 2.7. With the developed heat treatment profile, a carbon content of 0.153 wt.%, an oxygen content of 0.457 wt.% and a nitrogen content of 0.078 wt.% were measured.

**Figure 9:**
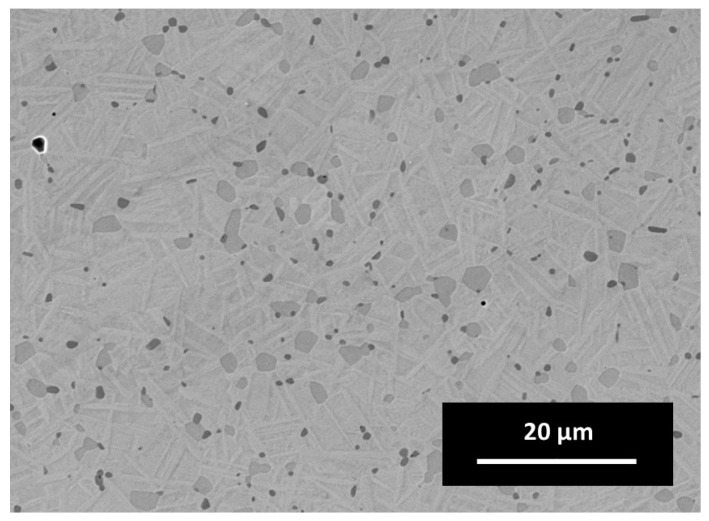
Microstructure of an as-sintered FFF sample using SEM; surface was not etched.

**Figure 10:**
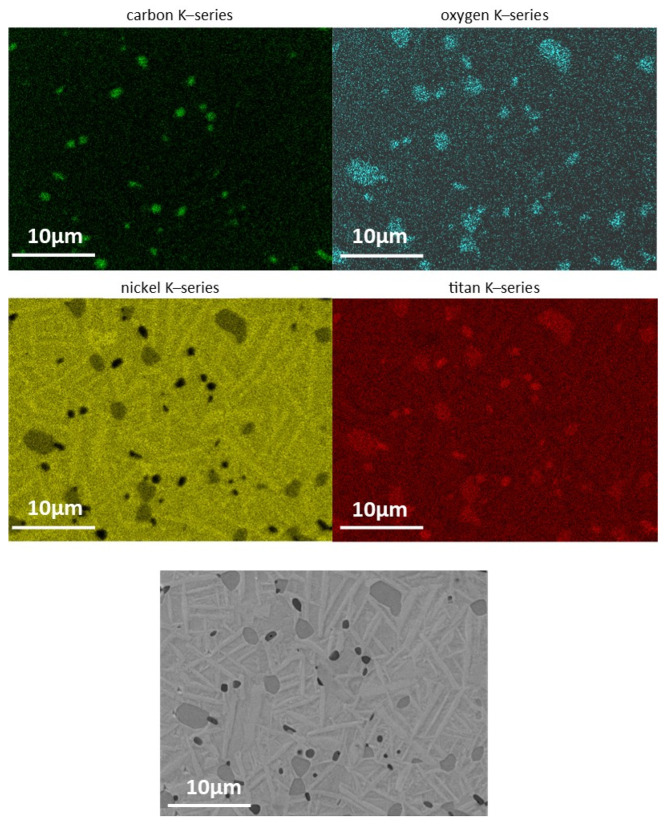
EDS mapping of an as-sintered FFF specimen.

Examining the microstructure in Figure 9, the dark gray Ti-carbo-oxides (Ti(C,O)) in between the typical rod-like NiTi grains are obvious. The light gray islands between the NiTi grains are oxygen- and carbon-containing NiTi alloys, which was confirmed using energy dissipative X-ray Spectroscopy (EDS). The EDS mapping containing C, O, Ni and Ti is illustrated in Figure 10. Most of the impurities are present as precipitates in the microstructure.

The most evident phases in this system are presented in the work of Carreira et al. [6]. This assumption is based on the fact that hydrocarbons (polymers) are also used and identified as the main source of impurities in this reference. Due to the additional oxygen and nitrogen in the binder’s amide group, the quantity can vary. However, nitrogen compounds are not detected in the microstructure, which is proven by the EDS point scan. It is expected that these carbides and oxides change the transition temperatures. It is clear that the heat treatment to reach a carbon free microstructure was not optimal and depends on the impurities of the initial powder, as well as the impurities introduced by the polyamide-like thermoplastic binder.

**Figure 11:**
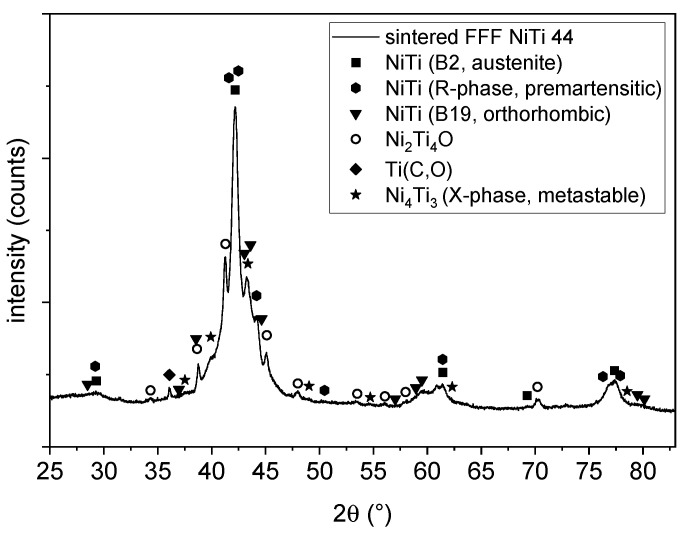
XRD analysis of the sintered sample showing a mixture of different phases. Next to the Ni-Ti phases in different modifications, Ni_2_Ti_4_O and Ti(C, O) also appear in the sample.

The phase composition of the sintered sample (see Figure 11) shows a mixture of different phases of NiTi. The penetration depth was estimated to be around 5–10 µm in an area of approximately 2 × 2 mm^2^. The main peak shows both the austenitic B2 phase as well as a pre-martensitic R phase [31]. Additionally, there are some reflections of an orthorhombic B19 phase. This phase was reported as intermediate phase in doped NiTi phases at the martensitic transformation [32]. Above this, some unknown reflection could occur by a metastable X phase [33]. The phase composition of the NiTi alloy suggests an intermediate state of the transformation from the austenitic (B2) to the martensitic phase (B19’). This may be due to the presence of different impurities of the sample as mentioned above. There are some secondary phases of Ni_2_Ti_4_O and a Ti(C, O) phase, which can also be seen clearly in the EDS mapping in Figure 9.

**Figure 12:**
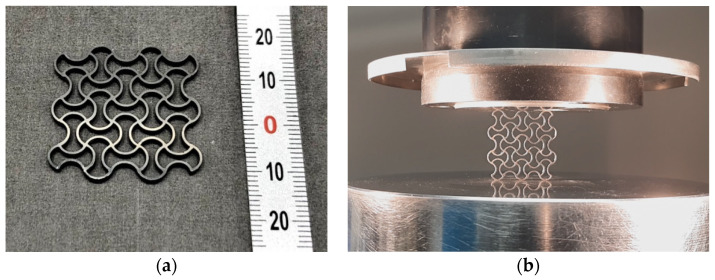
(**a**) Sintered auxetic component, wall thickness 0.5 mm. (**b**) Sample in cyclic compression test (video in Supplementary Materials).

The sintering results of the auxetic structures are shown in Figure 12. Due to the property of auxetic structures to exhibit a negative Poisson’s ratio, the component is also able to contract horizontally when compressed vertically. This is visualized in the Supplementary Materials by a video.

**Figure 13:**
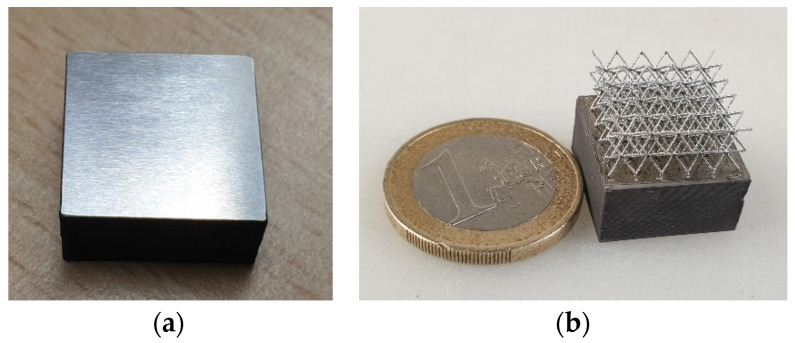
NiTi fabricated samples: (**a**) Substrate of 17 × 17 × 10 mm^3^ produced by FFF technology and (**b**) Hybrid structure including LPBF part (bcc lattice structure of 12 × 12 × 6 mm^3^) built on FFF part.

**Figure 14:**
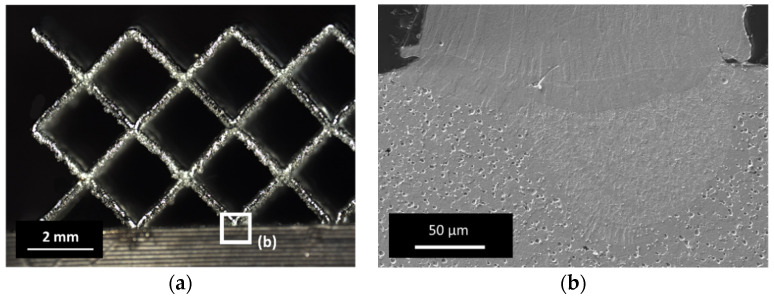
(**a**) Micrograph of filigree bcc lattice structure (Φ 300 µm); (**b**) SEM micrograph of the interface of FFF part (lower part) and LPBF lattice structure (upper part), surface was etched after Kroll.

The hybridization of a ground sample with a filigree bcc lattice is illustrated in Figure 13 and Figure 14. The result shows that a strong connection of both sections can be obtained using the parameters mentioned above. The microstructure of the etched interface shown in Figure 14b confirms the formation of a good material bond, where no systematic cracks are found.

The heat-affected zone penetrated the surface of the FFF component significantly, which indicates a good material bond. The porosity in the lower FFF section was caused by Kroll etching. The carbide and oxide grain boundary phases were attacked and dissolved, leaving holes. It can be assumed that an appropriate parameter window was found to hybridize sintered FFF samples using this approach.

## 4. Discussion

The fine powder fraction used for FFF has two main effects that degrade the overall performance. First, the sinter activity is high, which results in relatively short sintering times and a residual porosity as low as 1%. This is a very good value from a sintering point of view and sufficient for most applications. The extent to which the porosity affects the fatigue behavior of pseudoelastic components potentially subjected to high deformation in service remains to be seen. Secondly, the high powder surface favors the absorption of impurities. Examining the impurity values of the sintered parts more closely shows that they deviate significantly from the target values of the specification. According to the standard specification for wrought nickel–titanium Shape Memory Alloys for medical devices and surgical implants (ASTM 2063), the limits for nickel–titanium Shape Memory Alloys are 0.04 wt.% for oxygen and carbon and 0.005 wt.% for nitrogen. The measured impurity values of 0.153 wt.% (C), 0.457 wt.% (O) and 0.078 wt.% (N) are therefore too high and change the stoichiometric ratio of the alloy. One source of the impurities is the thermoplastic binder. 

The polyamide-based binder system obtains nitrogen and oxygen due to the amide group between the carbon-containing main chains. Some residuals of these elements remain in an inert thermal debinding phase, forming precipitations at higher temperatures. This binder system was carefully chosen because of its excellent processing properties and its two-stage debinding. During solvent debinding, the short-chain polymers of the blend are extracted and thus the excess carbon is reduced but not completely removed before thermal treatment. 

However, micrographs from EDS and XRD analysis revealed that carbides such as Ti(C, O) and oxides such as Ni_2_Ti_4_O were formed. This strongly influences the transformation temperatures between the martensite and austenite phases and thus the mechanical behavior and the occurrence of pseudoelastic behavior of the material. The motivation for using the challenging NiTi material lies in its pseudoelastic property. This occurs when the service temperature is above the austenite finish temperature. This is the temperature at which the transformation to austenite is completed, when the material is heated. In the DSC measurements, the temperature ranges of the phase transformation can be determined from the peaks. In the as-sintered condition, the A_f_ temperature is 90 °C. This value is significantly higher than the target application temperature of 20 °C. Since the application temperature in this case lies in the temperature range of the phase transformation, only very weak pseudoelastic behavior can be expected from the material, which is probably not sufficient for technical use. With the subsequent heat treatment for homogenization annealing, the temperature range of the phase transformation was successfully narrowed. Moreover, A_f_ at 30 °C is closer to the target range of the application temperature. The thermal post-treatment of sintered components to adjust the properties should therefore be considered more closely in the future. Nevertheless, temperatures for A_f_ in the range of 15 °C (ASTM F 2633), or ideally below 0 °C, are targeted for technical use.

Although thermal post-treatment can improve the properties, we see the impurities as the main cause of the phase transformation ranges determined. Therefore, the main objective for further development is to reduce the impurities by further adjusting the heat treatment of the printed FFF parts. To optimize heat treatment in terms of carbon and oxygen content reduction, alternative debinding methods need to be considered. Combined oxidation and subsequent reduction cycles could contribute in particular to decarbonization. The use of oxygen getter materials such as molybdenum sheets to enclose the samples could reduce the oxygen content.

Concerning the manufacturing process, we showed that the production of NiTi parts by FFF is possible. Besides the need to reduce the impurities, as discussed above, the competitiveness of the process should be increased by improving material deposition strategies to reduce gussets between the deposited strands. Under a load, these voids are origins of fracture and have a significant impact on the strength of the component. Addressing this major problem is a subject for current developments in printer equipment and printing strategies. For example, by implementing optical systems to monitor material deposition, an FFF system could be enabled to automatically correct defects in the same layer. Furthermore, a deposition strategy with material kneading (or wiggle) movements of the nozzle is conceivable to close small gaps between the strands.

Although the equipment for Additive Manufacturing of NiTi via the FFF process is inexpensive, there are, at the moment, still considerable costs for heat treatment under inert gas or vacuum. The cost-effectiveness can be improved by service providers who heat-treat components in series using optimized equipment.

In the present hybridization case, NiTi material was intended to be used from the very beginning, promising a successful, homogeneous hybridization from a material point of view. The general motivation behind the hybridization of LPBF is to combine the technology’s advantages with the specific advantages of concurrent technologies and to overcome the current drawbacks of LPBF [34], such as the high local mechanical stresses caused by high stiffness changes in the transition from melt to solid in compact areas, which could ultimately generate cracks under adverse conditions. Hybridization with FFF could become a viable alternative when compact substrates need to be supplemented with filigree sections by means of LPBF.

A major drawback in hybridization with LPBF as a successive manufacturing step is that interfaces cannot be curved, but must be fully flat and aligned in the XY build plane to subsequently recoat and solidify powder layers on top. In the present case, the FFF substrate had been complexly ground, providing a plane-parallel fixation of NiTi FFF substrate in the LPBF machine (Figure 13a).

## 5. Conclusions

The presented work illustrates a complete process chain for the Additive Manufacturing of NiTi components via fused filament fabrication, as well as the addition of filigree structures of the same material on sintered parts using Laser Powder Bed Fusion. The filaments developed within this study are suitable for processing on various standard Additive Manufacturing devices. They offer excellent flexibility and strength despite a very high solid loading of 63 vol.%, exceeding typical densities of powder bed-based AM methods for instance. By using fine powders that are not suitable for LPBF (<15 µm), FFF can be considered economical, in addition to the use of cost-effective standard AM equipment. Nevertheless, the heat treatment, which is typical for sinter-based AM, is expensive and delicate, since pure inert or reducing atmospheres are needed to fully densify NiTi compacts.

With optimized manufacturing parameter settings, green parts can be produced without voids between the strands. 

This contribution complements the phenomenology of samples made via FFF, consisting of intermetallic NiTi recently published in [6], and highlights the need for controlled heat treatment to reduce extraneous phases, such as carbides and oxides. By comparing the composition of the raw material powder and the sintered part, the thermoplastic binder was identified as the main source of influence for the formation of interfering carbides and oxides. It is assumed that the martensite-to-austenite transition temperature is directly influenced by the formation of such phases. Thus, the magnitude of the pseudoelastic effect may be reduced at room temperature, which must be evaluated in tensile tests in the future. The sintered parts exhibit a typical metal appearance and are structurally stable due to their low porosity of approximately 1% and the low level of manufacturing defects after parameter optimization. 

The addition of filigree structures (bcc lattice) by LPBF based on the same initial powder of different particle sizes (15–45 µm) results in a suitable solution for process hybridization. Observations of the interface show very good mechanical connectivity, which offers promising scope for, e.g., patient-specific personalization of medical devices or topology-optimized stiffness of structural components, by adding lattices using FFF substrates.

Additive Manufacturing of complex structures acting as a metamaterial, in combination with the outstanding reversible deformation characteristics of NiTi alloy, has added another degree of design freedom by the combination of FFF and LPBF AM technologies and contributes to paving the way towards truly programmable materials.

## Figures and Tables

**Table 1:** Parameters for AM of NiTi using FFF.

Parameter	Value
nozzle diameter (mm)	0.4–0.6
temperature (°C)	145–155
layer height (µm)	150–200
speed (mm/s)	30
temperature building platform	room temperature

## Supplementary Materials

The following are available online at: https://www.mdpi.com/article/10.3390/ma14164399/s1, Video S1: Cyclic test of auxetic structure.
